# Genistein binding protein targets in dental pathogens

**DOI:** 10.6026/973206300171109

**Published:** 2021-12-31

**Authors:** B Vivek Babu, AS Smiline Girija, J Vijayashree Priyadharsini

**Affiliations:** 1 Saveetha Dental College and Hospital, Saveetha Institute of Medical and Technical Science (SIMATS), Saveetha University, Chennai - 600077, India

**Keywords:** Genistein, phytochemicals, anti-microbial, dental pathogens, virulence proteins

## Abstract

Oral pathogens have created a menace in recent years due to biofilm formation and antimicrobial drug resistance. The current treatment strategy works well with antibiotics. However, constant use of antibiotics creates a selective pressure, which increases adaptability of the pathogens. Therefore, it is of interest to analyze the potential targets of genistein in dental pathogens using computer aided prediction tools.

## Background:

Antimicrobial agents have long been used to target pathogenic microorganisms. In many instances the antibiotics administered fail to act upon the pathogens due to increasing antimicrobial resistance. A potent antibiotic against drug resistant microbes is critical. Hence, an alternative measure has to be adopted to combat such menace in the clinical settings. Several dental pathogens have also been reported to harbor drug resistant genes, which tend to hamper the treatment procedure [1-2]. Alternative medicine using herbs and bioactive compounds from plants is of significance. Isoflavones are flavonoids which are found to exhibit antioxidant, antimicrobial, anti-inflammatory and anticancer properties. Several reports have confirmed the effect of isoflavones in preventing chronic inflammatory diseases [3]. One of the important isoflavone present in soy is genistein and it is a phytoestrogen. The biological activities of genistein include inhibition of inflammation, modulation of steroidal hormone receptors, promotion of cell death by apoptosis and regulation of metabolic pathways [4 - check with author]. Since these pathways are directly or indirectly related to inflammatory, metabolic diseases and malignant transformation, genistein is an excellent alternative for synthetic drugs used to treat such disorders. Genistein being a protein kinase inhibitor exhibits antibacterial activity by preventing the invasion of pathogenic bacteria in mammalian epithelial cells [5 - check with author]. Oh et al. showed the antimicrobial activity of genistein against a model organism for bacterial septicaemia Vibrio vulnificus. They found that genistein inhibited the adhesion of V. vulnificus to HeLa cells and prevented cell death [6]. Therefore, it is of interest to analyze the potential targets of genistein in dental pathogens using compter aided prediction tools.

## Materials & Methods:

### Strains used in the study:

The following strains available in the STITCH database were used for the study. Streptococcus mutans UA159, Enterococcus faecalis V583, Porphyromonas gingivalis ATCC 33277, Treponema denticola ATCC 35405 and Tannerella forsythia ATCC 43037.7

### Analysing protein interaction network:

STITCH is an exhaustive pipeline used for predicting the interactions between chemicals and proteins. The interactions are of two types (a) direct or physical and (b) indirect or functional associations which arise from data accumulated in the primary databases. The repertoire of proteins interacting with Porphyromonas gingivalis, Treponema denticola and Tannerella forsythia were used for predicting virulence [7]. The FASTA format of protein sequences was retrieved from the National Centre for Biotechnology Information (NCBI) [8].

### Prediction of functional class of interacting proteins:

VICMpred server is used for the classification of pathogenic microbial proteins into four major classes namely, virulence factors, information and storage processing, cellular process and metabolism. The principal virulence factors such as adhesins, toxins and haemolytic molecules are identified using the support vector machine (SVM) algorithm, which classifies proteins based on their amino acid composition and sequence pattern [9].

### Prediction of virulence properties of interacting protein:

The identification of virulent bacterial protein targeted by a drug or phytocompound helps in substantiating the antimicrobial activity of the compound. VirulentPred is a yet another SVM based method, used for automated prediction of virulent proteins based on the sequences (bioinfo.icgeb.res.in/virulent). The scores with positive predicted values are more often categorised into virulent protein and those with negative predicted values are categorised as avirulent proteins [10].

### Prediction of B-cell epitopes in the virulence proteins:

Epitopes are small regions on the antigens recognized by antibodies. Identification of B-cell epitopes on the virulence proteins identified adds advantage to the phyto compound selected. BepiPred 2.0 server was used to identify the peptide epitopes on the virulent proteins. The peptide molecules, which scored above a threshold greater than 0.5 are predicted to be part of the epitope and are, colored yellow in Figure 2 [11-12].

### Prediction of sub-cellular localization of proteins:

The identification of the subcellular localization of virulence proteins is of prime importance as the efficiency of the compound lies in target identification. Cell surface proteins are readily targeted, whilst, the cytoplasmic or nuclear proteins need proper drug delivery systems to target the protein of interest. Hence, PSORTb V.3.0 was used for identification of sub-cellular location of virulence proteins [12].

## Results and Discussion:

**Figure 1:**
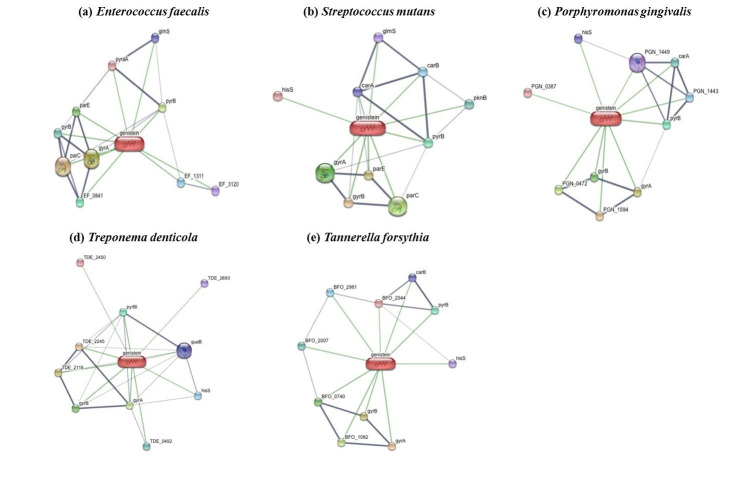
Interaction of genistein with the protein repertoire of common dental pathogens. (a) Enterococcus faecalis, (b) Streptococcus mutans, (c) Porphyromonas gingivalis, (d) Treponema denticola, (e) Tannerella forsythia

**Figure 2:**
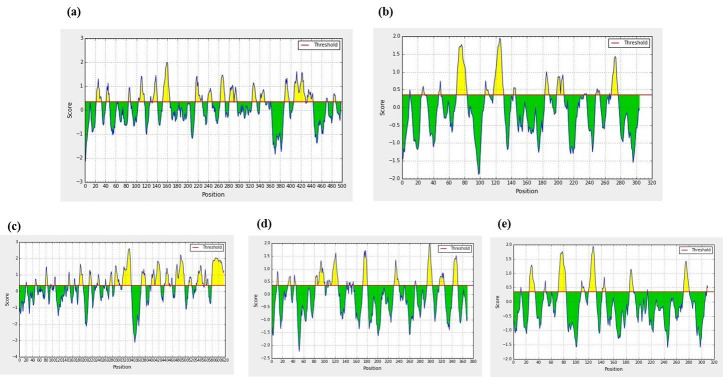
Predicted epitopes on the virulence protein (a) Inosine-5'-monophosphate dehydrogenase and (b) aspartate carbamoyltransferase of Porphyromonas gingivalis, (c) Serine/threonine protein kinase of Streptococcus mutans, (d) sensor histidine kinase/response regulator of Treponema denticola, (e) aspartate carbamoyltransferase catalytic chain of Tannerella forsythia

The computer aided prediction tools were used to find the potential targets of genistein in Enterococcus faecalis, Porphyromonas gingivalis, Streptococcus mutans, Treponema denticola and Tannerella forsythia of which some of them were virulence. Software like STITCH, VICMPred, PSORTb and BepiPred were used to identify the drug protein interactions, virulent nature of the proteins, subcellular localization and putative epitopes in the proteins identified (Figure 1). Inosine-5'-monophosphate dehydrogenase and aspartate carbamoyltransferase of Porphyromonas gingivalis, Serine/threonine protein kinase of Streptococcus mutans, Sensor histidine kinase/response regulator of Treponema denticola, Aspartate carbamoyltransferase catalytic chain of Tannerella forsythia were identified as virulence factors (Table 1 - see PDF). These virulence factors were involved in metabolism and cellular processes. Enzymes involved in DNA replication processes such as gyrase and topoisomerase were targeted by genistein in all the five species of bacterial pathogens. The subcellular location of virulence proteins was in the cytoplasm, with an exception of serine/threonine protein kinase and sensor histidine kinase/response regulator, which were found in the cytoplasmic membrane (Table 2 - see PDF). The location of drug targets is very vital in drug designing, pharmacokinetics and dynamics of the compound used for analysis. Moreover, putative peptide epitopes were identified in the virulence proteins targeted by genistein (Figure 2). These epitopes are antigen-determining sites on the virulence proteins identified by the host immune system. Targeting an epitope on the virulence protein is known to improve the efficacy of the compound. Bacteria associated with dental diseases are present as a polymicrobial community. S. mutans, E.faecalis, Lactobacillus salivarius, L. plantarum, Actinomyces naeslundii are a few bacteria, which colonize and form biofilm. Although there are mechanical and chemical methods available to get rid of these pathogens, complex structures like biofilms are difficult to manage due to their refractoriness to antibiotic treatments. The existing procedures at times exhibit adverse effects such as allergic reactions, tissue damage etc., also, they have not guaranteed complete removal of pathogenic bacteria [13]. Numerous compounds have been tested from bacteria, plants and other sources to identify the best compound with an efficacy to target and eradicate pathogens leaving the host tissues unharmed [14][15]. Antibiotic dressing employing triple antibiotic paste is usually done to facilitate endodontic regeneration [16]. An acute or chronic exposure to such antibiotics will eventually lead to selective pressure that aids in the transformation of antibiotic susceptible strain into antibiotic resistant strains. Hence, such exposure can be avoided by use of alternative compounds, which do not impose selective pressure on pathogens. Genistein and derivatives of ginestein have been identified in Pterospartum tridentatum, a plant used in folk medicine to treat diseases related to inflammatory processes. P. tridentatum extract was shown to exhibit the highest antibacterial activity in a dose dependent manner against methicillin-resistant (MRSA) and methicillin-sensitive Staphylococcus aureus MSSA. This antibacterial activity is due to high content of flavonols, flavones and isoflavones, which act synergistically to exert their role against this type of bacteria [17,18]. The antibacterial effect of genistein upon pathogens like Escherichia coli, Shigella sonnei, and Staphylococcus aureus as well as Klebsiella pneumoniae and the non‐pathogenic organism, Bacillus anthracis (Sterne) is known. Subsequently, significant reductions in colony forming units were recorded for S. aureus and B. anthracis when cultured in the presence of 100-µM genistein. Modified derivatives of genistein were screened for their antibacterial and antifungal activities by MTT method against gram positive bacteria such as Bacillus subtilis, Staphylococcus aureus, gram negative bacteria such as Escherichia coli, Pseudomonas fluorescens and fungal species Trichophyton rubrum, Candida albicans. Promising results supports the design of the present study [19]. A similar study by Li et al. used deoxybenzoin derivatives from genistein against B. subtilis, E. coli, P. fluorescence, S. aureus, A. niger, C. albicans and T. rubrum. Most of the derivatives tested showed significant antibacterial effects [20]. However, information is not available on the molecular mechanisms related to the antimicrobial activity of genistein. A few studies have provided evidence on the inhibitory role of the compound. Verdrengh et al. showed the growth inhibition of S. aureus that is mediated by inhibition of topoisomerase IV [21]. Data shown here is consistent with the above report where genistein was found to act on a common target DNA topoisomerase IV A and B. Thus, we report the data on the potential targets of genistein in dental pathogens using computer aided prediction tools. The virulence-encoded proteins were differentiated using specific computational tools and the epitopes associated with those proteins.

## Conclusion:

We document the potential targets of genistein in dental pathogens using computer aided prediction tools.
